# TGFBI expression is associated with a better response to chemotherapy in NSCLC

**DOI:** 10.1186/1476-4598-9-130

**Published:** 2010-05-28

**Authors:** Marta Irigoyen, María J Pajares, Jackeline Agorreta, Mariano Ponz-Sarvisé, Elisabeth Salvo, María D Lozano, Ruben Pío, Ignacio Gil-Bazo, Ana Rouzaut

**Affiliations:** 1 Division of Oncology, Center for Applied Medical Research, Universidad de Navarra, Pío XII 55, Pamplona, 31008, Spain; 2 Department of Oncology, Clínica Universidad de Navarra, Pío XII 36, Pamplona, 31008, Spain; 3 Department of Pathology, Clínica Universidad de Navarra, Pío XII 36, Pamplona 31008, Spain

## Abstract

**Background:**

Lung cancer is one of the most prevalent neoplasias in developed countries. Advances in patient survival have been limited and the identification of prognostic molecules is needed. Resistance to treatment is strongly related to tumor cell adhesion to the extracellular matrix and alterations in the quantity and nature of molecules constituting the tumor cell niche. Recently, transforming growth factor beta-induced protein (TGFBI), an extracellular matrix adaptor protein, has been reported to be differentially expressed in transformed tissues. Loss of TGFBI expression has been described in several cancers including lung carcinoma, and it has been suggested to act as a tumor suppressor gene.

**Results:**

To address the importance of TGFBI expression in cancer progression, we determined its expression in NSCLC clinical samples using immunohistochemistry. We identified a strong association between elevated TGFBI expression and the response to chemotherapy. Furthermore, we transiently over-expressed and silenced TGFBI in human NSCLC cell lines. Cells over-expressing TGFBI displayed increased sensitivity to etoposide, paclitaxel, cisplatin and gemcitabine. We observed that TGFBI-mediated induction of apoptosis occurred through its binding to αvβ3 integrin. We also determined that full-length TGFBI did not induce caspase 3/7 activation but its proteolytic fragments that were < 3 kDa in size, were able to activate caspase 3, 7 and 8. This pro-apoptotic effect was blocked by anti-αvβ3 integrin antibodies.

**Conclusions:**

The results shown here indicate that TGFBI is a predictive factor of the response to chemotherapy, and suggest the use of TGFBI-derived peptides as possible therapeutic adjuvants for the enhancement of responses to chemotherapy.

## Background

More than a century ago Paget proponed his "seed and soil hypothesis" in which the tumor environment (*soil*) was just as important as cancer cell itself (*seed*) [1,2]. Since then, increasing attention has been devoted to the behavior of stromal cells and to the tumor microenvironment. In fact, it is now well established that minor alterations in one of the constituents of the tumor niche may cause dramatic reorganizations of the whole system [3]. Hence, binding of cancer cells to the extracellular matrix (ECM) influences cell behavior and, ultimately, tumor progression [4].

A panoply of ECM receptors and binding proteins have been associated with tumor adhesion and cancer progression [5,6]. One of these molecules is TGFβ1-induced protein (TGFBI), which is also known as keratoepithelin or βIg-h3. This protein was first described in TGFβ1-treated A549 non-small cell lung cancer (NSCLC) cells and was soon associated with corneal dystrophies, which are the only known pathological manifestations of genetic mutations in TGFBI [7,8]. This 68-kDa protein contains four conserved fasciclin-1 (FAS1) domains and a carboxyl-terminal Arg-Gly-Asp (RGD) integrin-binding sequence. TGFBI mediates integrin binding to ECM proteins such as collagen, laminin and fibronectin. TGFBI binding to integrins has been related to the activation of cell proliferation, adhesion, migration and differentiation [9]. This protein is upregulated in human tumors of the colon [10], renal [11,12], pancreas [13] and in neuroblastoma [14], whereas it is down-regulated in breast cancer [15]. TGFBI has conflicting roles in cancer progression. Depending on the tissue, TGFBI functions as a promoter or suppressor of cancer growth. For example, overexpression of TGFBI in renal, pancreatic or colon carcinoma cells induces cell migration and increased metastatic potential [12,13,16]. Conversely, Zhang et al. demonstrated that TGFBI-/-mice were susceptible to both spontaneous and 7,12-dimethylbenz(α)anthracene-induced skin tumors [17]. In relation to lung cancer, loss of TGFBI expression has been described in asbestos and radiation-exposed human bronchial epithelial cells [18,19] and in human lung cancer samples [20,21]. 

In the present work, we described an association between TGFBI expression and the response to chemotherapy in NSCLC. In order to characterize the molecular mechanisms underlying this association, we examined the effects of TGFBI over-expression and inhibition in NSCLC cells on the induction of apoptosis after chemotherapy. Additionally, we evaluated the effect of soluble proteolytic fragments of TGFBI on tumor cell survival.

## Results

### High TGFBI expression in NSCLC samples correlates with response to chemotherapy

Loss of TGFBI expression has been associated with the tumorigenic phenotype of several carcinomas including lung cancer [[20], and Additional file 1]. It has also been correlated with enhanced sensitivity to paclitaxel and etoposide in ovarian carcinoma [22] and a lung adenocarcinoma cell line [20], respectively.

**Figure 1:**
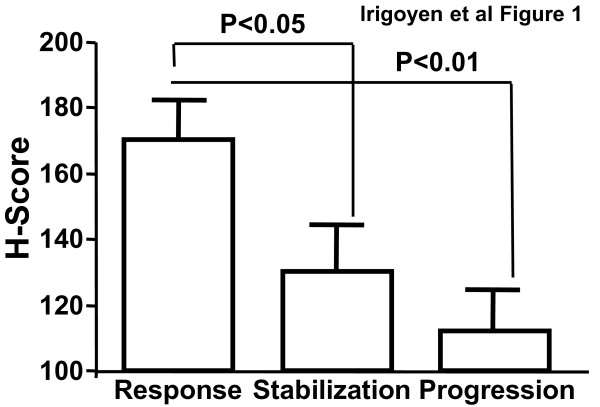
**High TGFBI expression is associated with a positive response to chemotherapy in NSCLC samples**. Mean H-score expression levels of TGFBI were determined by immunohistochemistry (n = 47) in a series of NSCLC biopsies obtained before chemotherapy. Statistical differences between response and stabilization and response versus progression were analyzed using Kruskal-Wallis and pair-wise Mann-Whitney U tests. *p < 0.05; **p < 0.01.

In order to determine if loss of TGFBI expression correlated with the chemotherapeutic response in clinical samples, we retrospectively analyzed pre-chemotherapy TGFBI protein expression in a series of 47 stage IV NSCLC samples by immunohisto-chemistry. TGFBI expression was significantly (*p *= 0.003) higher in patients who responded to chemotherapy (average TGFBI H score = 170) compared to patients whose cancer progressed (average score = 110) or who had stable disease (average score = 120) (Figure 1). We did not observe any correlation between TGFBI expression and other clinico-pathological parameters analyzed such as gender, age or tumor histology. Detailed information regarding TGFBI expression in each tumor sample, patient outcome and the type of chemotherapy administered is provided in Additional file 2.

We also performed Kaplan-Meier analysis of overall survival on the same series of patients. Data shown in Additional file 3 illustrate that patients expressing higher levels of TGFBI show a trend towards better survival (p = 0.053). This data, in spite of not being significant, suggests its possible use as a candidate prognostic factor. Nevertheless, analyses performed in greater number of samples should be made to definitively test this.

### TGFBI over-expression contributes to NSCLC responses to chemotherapy

**Figure 2:**
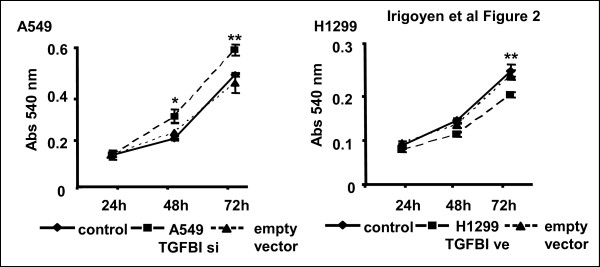
**Changes in TGFBI expression affects cell viability**. Cell viability of transfected NSCLC cells was determined by neutral red staining 24, 48 and 72 h after transfection with a TGFBI siRNA plasmid or a TGFBI expression vector. Transfection with empty vector was used as a negative control for all experiments. Three independent experiments were performed. Statistical comparisons of the differences between control and transfected cells were performed using Student *t*-test (* *p *< 0.05; **p < 0.01).

To identify the mechanism underlying the role of TGFBI in tumor cell response to chemotherapy, we transiently over-expressed TGFBI in the metastatic NSCLC cell line NCI-H1299, which has a low basal expression of this protein (H1299-TGFBIve). Additionally, we silenced TGFBI in non-metastatic NSCLC NCI-A549 cells, which express high basal levels of this protein (A549-TGFBIsi). Basal expression and the efficiency of TGFBI over-expression and silencing are shown in Additional file 4. Using this experimental approach, we determined the effects of TGFBI expression on cell viability using the neutral red assay. TGFBI silencing in A549 cells increased cell viability while over-expression in H1299 cells diminished survival (Figure 2).

**Figure 3:**
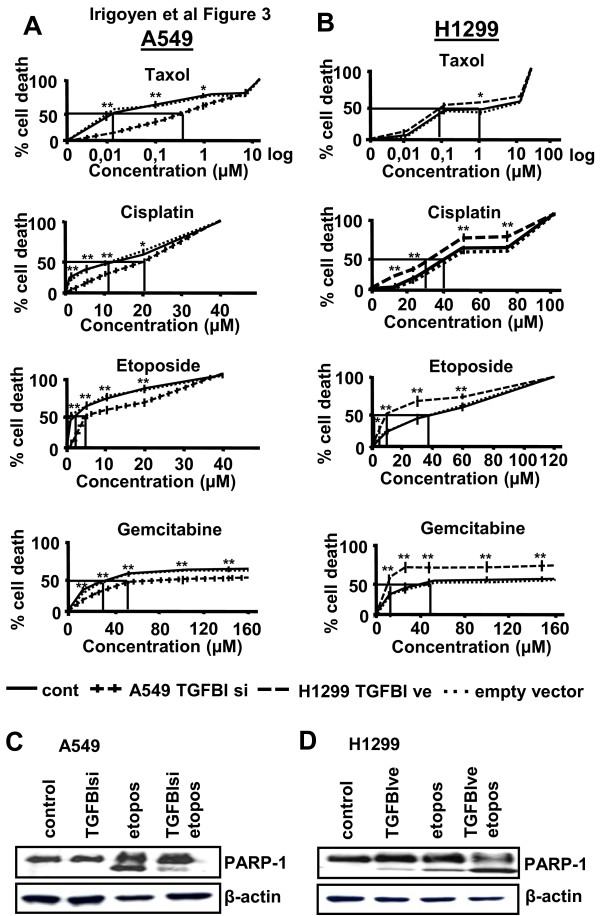
**TGFBI sensitizes NSCLC cells to chemotherapy**. A549 cells transfected with TGFBI siRNA plasmid (TGFBIsi) (A), and H1299 cells transfected with TGFBI expression vector (TGFBIve) (B), were exposed to increasing amounts of different cytotoxic agents for 48 h and cell viability was measured. Three independent experiments were performed. Comparisons to analyze statistical differences between control and transfected cells were performed using Student *t*-test (* *p *< 0.05; ** *p *< 0.01). IC_50 _for each experimental condition is indicated. (C, D) PARP-1 cleavage was detected by Western blot analysis of total protein extracts derived from A549-TGFBIsi (C) and H1299-TGFBIve (D) transfected cells treated with etoposide for 48 h. One representative experiment out of three is shown.

Since cells that over-expressed TGFBI showed reduced viability, we postulated that they may be more sensitive to chemotherapy than cells not expressing this protein. To explore this, TGFBI over-expressing (H1299-TGFBIve) or silenced (A549-TGFBIsi) NSCLC cells were treated with paclitaxel, etoposide, cisplatin or gemcitabine and measured their IC_50_. A549-TGFBIsi cells displayed a higher IC_50 _for all drugs assayed compared to treated non-transfected or empty vector-containing cells, with more prominent effects observed in cells treated with paclitaxel and etoposide (Figure 3A). On the contrary, H1299-TGFBIve cells displayed a significant decrease in their IC_50 _(Figure 3B) compared to control cells. We measured PARP cleavage in A549-TGFBIsi and H1299-TGFBIve cells by Western blot analysis after their exposure to etoposide. After etoposide treatment, PARP cleavage in A549-TGFBIsi cells was reduced (Figure 3C), whereas H1299-TGFBIve displayed higher PARP activation (Figure 3D). Therefore, we confirmed that TGFBI expression augmented cell death in NSCLC cells after exposure to chemotherapeutic drugs. Interestingly, the increase observed in cell death following chemotherapy occurred independently of the drug used and, therefore, of the cellular process targeted by these compounds (cytoskeleton remodeling and cell proliferation), which suggests that elevated expression of TGFBI has a direct pro-apoptotic effect on NSCLC cells. To test these, we measured cell viability in non treated NSCLC cells in which TGFBI had been silenced (A549 NSCLC cells) or over-expressed (H1299 NSCLC cells). The results obtained confirmed our hypothesis: those cells that had TGFBI silenced were more viable, while on the contrary, if this protein was over-expressed they presented reduced cell viability (Additional file 5).

### NSCLC cells exposed to recombinant soluble TGFBI displayed an increased susceptibility to chemotherapy

**Figure 4:**
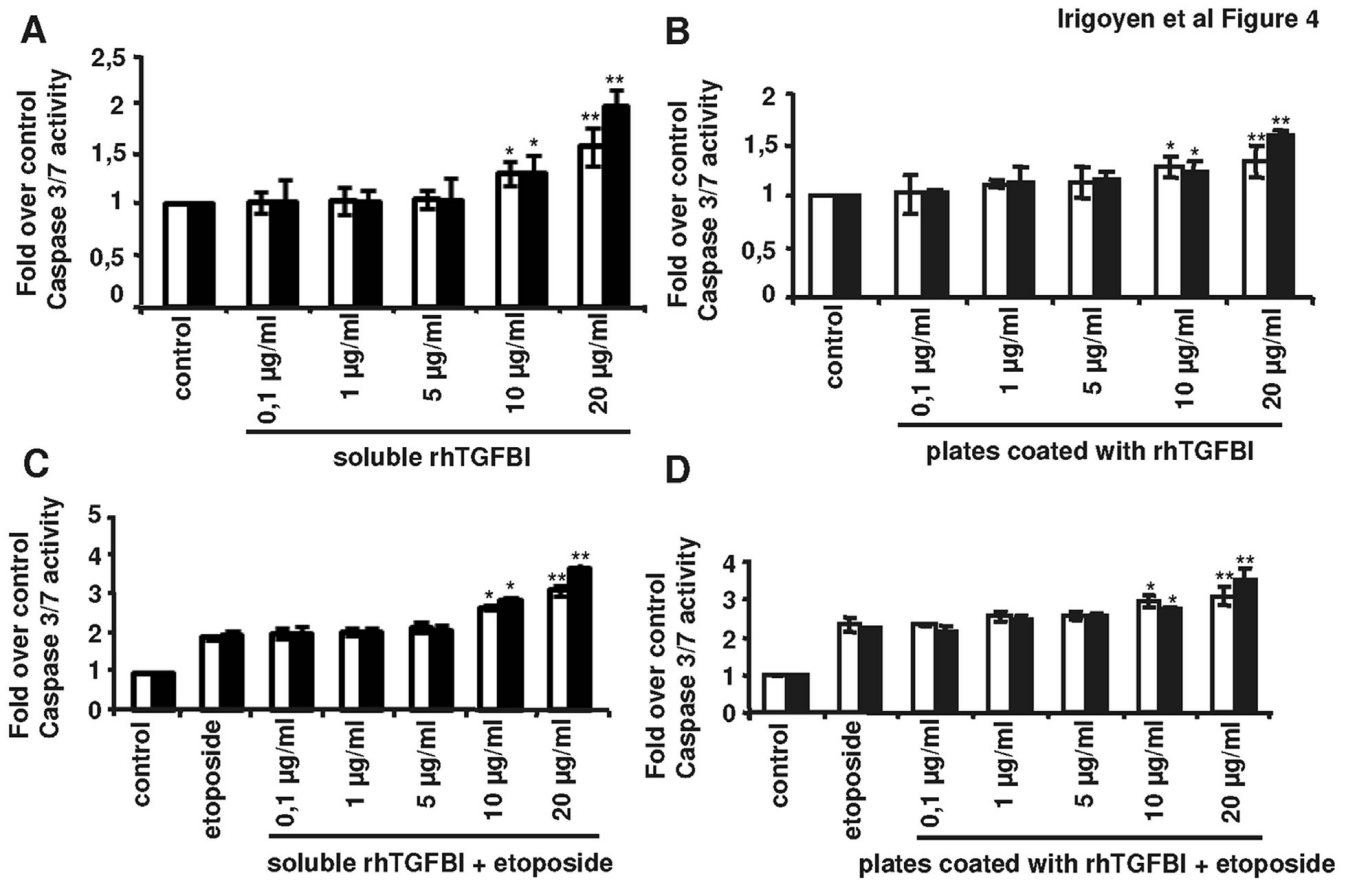
**High concentration of rh-TGFBI protein increases cell death and potentiates NSCLC responses to chemotherapy**. Caspase 3/7 activity was measured in A549 (white histograms) and H1299 (black histograms) NSCLC cells seeded onto non coated 96-well plates and further treated with increasing amounts of rh-TGFBI (A), or seeded onto 96 well plates coated with increasing amounts of rh-TGFBI (B), using the caspase 3/7 Glo-assay from Promega. (C and D) Caspase 3/7 activity of 24 h etoposide-treated A549 (5 μM) and H1299 (50 μM) cells seeded onto non coated 96-well plates and further exposed for 24 h to increasing amounts of rh-TGFBI previous to etoposide exposure (C) or seeded onto 96-well plates coated with increasing amounts of rh-TGFBI before exposure to etoposide (D). Statistical analyses were performed using Student *t*-test to compare caspase 3/7 activity in treated cells to non-treated cells (* *p *< 0.05; **p < .01).

Given that TGFBI is an extracellular protein, we next sought to ascertain if the observed effects were reproducible by the addition of exogenous recombinant TGFBI (rh-TGFBI). We measured caspase 3/7 activity in A549 and H1299 NSCLC cells after being exposed to increasing amounts of rh-TGFBI. As shown in Figure 4A, no pro-apoptotic effect was observed in response to low concentrations of recombinant TGFBI but, following exposure to 20 μg/mL rh-TGFBI, a 1.4- and 1.7-fold increase in caspase 3/7 activity was seen in A549 and H1299 cells, respectively. We also observed the same results when the equivalent amounts of recombinant protein were used to coat 96-well plates before seeding the carcinoma cells onto them (Figure 4B).

To determine if NSCLC binding to soluble recombinant TGFBI also contributed to improved responses to chemotherapy, we analyzed caspase 3/7 activation in NSCLC cells exposed to 20 μg/mL rh-TGFBI and treated with etoposide at a concentration of 5 μM and 50 μM for A549 cells and H1299 cells, respectively. NSCLC cells treated with etoposide in the presence of recombinant TGFBI displayed higher caspase 3/7 activity compared to cells treated with the chemotherapeutic drug alone (Figure 4C). As expected, when seeding A549 and H1299 cells onto TGFBI coated 96-well plates, an increased response to etoposide was also induced (Figure 4D).

### TGFBI-induced cell death is mediated through interaction with the αvβ_3 _integrin

**Figure 5:**
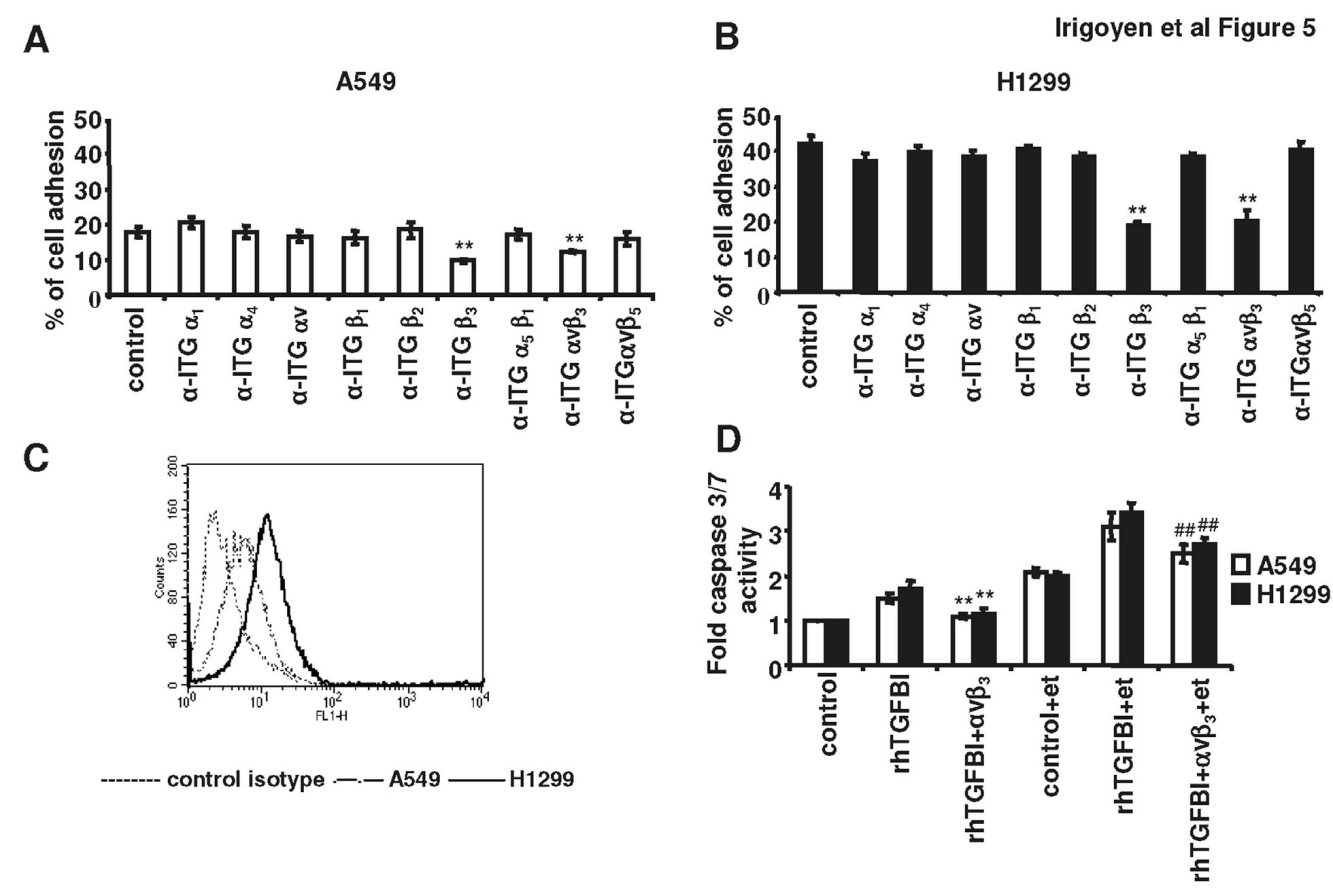
**TGFBI influences NSCLC susceptibility to chemotherapy through binding to the αvβ_3 _integrin**. Adhesion of calcein-labeled A549 (A) and H1299 (B) NSCLC cells to rh-TGFBI coated (1 μg/mL) 96-well plates was evaluated in the presence of antibodies against several integrins. Statistical analyses were performed using Student *t*-test and comparing treated cells to control cells (**p < 0.01). (C) Flow cytometry analysis of basal αvβ3 integrin expression in A549 cells (dashed histogram), H1299 cells (line histrogram) or isotype control (dotted histogram). (D) Caspase 3/7 detection in A549 and H1299 cells seeded onto non-coated 96 well-plates, pre-incubated for 1 h with anti-αvβ3 monoclonal blocking antibody, exposed to rh-TGFBI (20 μg/mL) for 24 h and further treated with etoposide at the IC_50 _(5 μM for A549 cells and 50 μM for H1299 cells) for additional 24 h. Statistical comparisons were performed using Student *t*-test. **p < 0.01 for the comparison of the activity control cells versus integrin blocked cells. ##*p *< 0.01 for the comparison of the activity of etoposide-treated cells versus cells treated in the presence of the anti-integrin blocking antibody.

Once we demonstrated that TGFBI induced apoptosis in our system, we next wanted to ascertain which cell surface receptor was required. Integrins are the only type of cellular receptors that have been shown to mediate TGFBI signaling. To determine which integrin was involved in TGFBI binding to NSCLC cells, we performed cell adhesion assays using NSCLC cells cultured onto plate-bound rh-TGFBI (1 μg/mL) in the presence of a variety of anti-integrin blocking antibodies. The adhesion of A549 and H1299 cells to rh-TGFBI was blocked only in the presence of the anti-αvβ3 integrin monoclonal antibody (Figures. 5A and 5B). A noticeable difference in basal cell adhesion to rh-TGFBI was observed between A549 and H1299 cells, the former showing lower adhesion than the latter. When we determined by flow cytometry the expression levels of the integrin αvβ3 on the surface of both cell lines, we observed that A549 cells expressed lower αvβ3 integrin than H1299 cells. This is probably the cause of their lower adhesion to TGFBI coated plates (Figure 5C).

It has been previously shown that αvβ3 integrin mediates paclitaxel sensitivity of ovarian cancer cells that transiently expressed TGFBI [22]. Therefore, to determine if this integrin influences TGFBI-mediated NSCLC cell susceptibility to chemotherapy, we incubated NSCLC cells with a blocking antibody specific for αvβ3, 1 h before the addition of rh-TGFBI (20 μg/mL) for 24 h and an further exposure to etoposide (5 μM for A549 cells and 50 μM for H1299 cells) for additional 24 h. As shown in figure 5D, blocking αvβ3 integrin resulted in impaired TGFBI-induced caspase 3/7 activity in both the presence and absence of etoposide. All these results demonstrated that this integrin participates in TGFBI-mediated induction of NSCLC cell apoptosis.

### RGD peptides derived from TGFBI influence the susceptibility of NSCLC cells to chemotherapy through caspase 8 and caspase 3/7 activation

TGFBI contains a carboxy-terminal Arg-Gly-Asp (RGD) integrin binding sequence. Given that integrin-mediated binding of a cell to the ECM or to small soluble peptides can have contrasting effects on survival, with the latter resulting in survival and the former leading to cell death [23], we addressed whether TGFBI-derived RGD-containing peptides played a role in TGFBI-mediated apoptosis.

**Figure 6:**
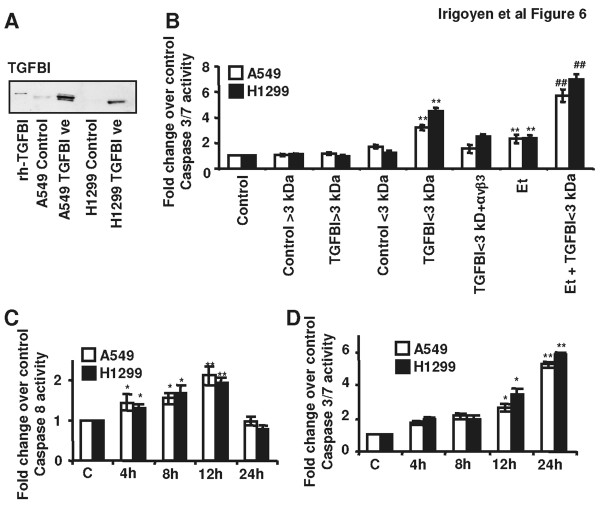
**Peptides derived from TGFBI mediate NSCLC cells response to chemotherapy and the induction of caspase 8 and caspase 3/7 activation**. (A) Western blot detection of proteolytic fragments of TGFBI in H1299 and A549 cells transiently transfected with the TGFBI expression vector. (B) Detection of caspase 3/7 activity in A549 and H1299 cells exposed for 24 h to cell supernatants derived from cultures of A549 and H1299 cells transiently transfected with the TGFBI expression vector. **Control**: non-treated cells; **Control > 3 kDa**: NSCLC cells exposed to supernatants from non-transfected cell cultures containing fragments larger than 3 kDa in size; **Control < 3 kDa**: NSCLC cells exposed to supernatants from non-transfected cell cultures containing fragments smaller than 3 kDa in size; **TGFBI > 3 kDa**: NSCLC cells exposed to supernatants from TGFBI-transfected cells containing fragments larger than 3 kDa in size; **TGFBI < 3 kDa**: NSCLC cells exposed to supernatants from TGFBI-transfected cells containing fragments smaller than 3 kDa in size; **αvβ3**: cells blocked with anti-αvβ3 blocking antibody 1 h before the addition of the supernatant; **Et**: Cells exposed to IC_50 _etoposide for 24 h. Statistical analyses were performed using Student *t*-test to compare caspase 3/7 activity in control cells (** *p *< 0.01) or etoposide treated cells (## *p *< 0.01) to the caspase activity in cell cultures treated with supernatants. (C) Caspase 8 and (D) caspase 3/7 detection in A549 and H1299 cells maintained for different time-periods in the presence of TGFBI < 3 kDa supernatant. Statistical analyses were performed using Student *t*-test and the caspase activity of treated cells was compared to that of untreated cells. (*p < 0.05; **p < 0.01).

The supernatants from the culture of A549 and H1299 NSCLC cells transiently transfected with the TGFBI expression vector were collected 24 h after transfection, and filtered through a membrane with 3 kDa molecular weight cut-off. Two different cell supernatants were obtained (TGFBI < 3 kDa and TGFBI > 3 kDa). They were separately added to A549 and H1299 cell cultures and, 24 h later, caspase 3/7 cleavage was evaluated to determine if TGFBI-derived peptides influenced cell apoptosis. NSCLC cells over-expressing TGFBI generated a TGFBI-specific band, which was smaller than the full-length 68 kDa recombinant protein, indicating that the TGFBI protein was digested (Figure 6A). The results presented in figure 6B demonstrated that only the smaller TGFBI-derived fragments (TGFBI < 3 kDa) induced caspase 3/7 activity in both A549 and H1299 NSCLC cells, while supernatants containing proteins larger than 3 kDa in size, or those obtained from non-transfected cells, had no effect on caspase activity. Even more, cell supernatants containing proteins larger than 3 kDa were unable to enhance the etoposide-induced increase in caspase 3/7 activation, whereas those supernatants containing smaller TGFBI fragments increased caspase activation similar to that induced by treatment of NSCLC cells with recombinant TGFBI protein. To support these observations, we exposed H1299 cells to short synthetic peptides derived from the RGD region of the TGFBI protein described by Kim and co-workers [24]. Results shown in Additional file 6 demonstrated that peptides containing the full RGD sequence induced cell death while a RGE mutant did not induce it.

Additionally, we found that the pro-apoptotic effects of the TGFBI < 3 kDa supernatants were abrogated by pre-incubating the NSCLC cells with an anti-αvβ3 integrin blocking antibody. We further confirmed the role of the αvβ3 integrin in TGFBI induced cell death by transfection of H1299 with an anti-β3 integrin sh-RNA plasmid and further exposing these cells to 3 < kDa supernatants derived from TGFBI over-expressing cells for 48 h. Caspase 3/7 activity remained basal in β3 integrin silenced cells whereas it was induced in H1299 cells transfected with a scramble-shRNA (Additional file 7).

It has been previously demonstrated that caspase-8 is recruited to a complex containing unligated αvβ3 integrins, where it is activated and mediates apoptosis [25]. In order to determine whether this caspase pathway is activated by TGFBI fragments, we measured caspase 8 activation in NSCLC cells exposed to supernatants containing TGFBI fragments < 3 kDa. Caspase-3/7 activation peaked 24 h after exposure to cell supernatants, while caspase-8 activation peaked 12 h earlier, which was expected due to its known previous activation in the caspase activation cascade (Figure 6C and 6D).

All of these results demonstrated that proteolytic fragments derived from TGFBI induced cell death through binding to the αvβ3 integrin on the surface of NSCLC cells and the subsequent activation of caspases 8 and caspase 3/7 signaling. These findings may explain the important association observed between elevated TGFBI expression and the response to chemotherapy found in NSCLC clinical biopsies.

## Discussion

Non-small cell lung cancer constitutes the largest subgroup of lung cancers, accounting for approximately 80% of the diagnosed cases of lung cancer. Most patients with NSCLC are diagnosed with locally advanced or metastatic disease and have very low survival expectancy [26,27]. The existing treatment options for these patients have considerable limitations in effectiveness and safety. Current efforts have focused on discovering markers that are predictive of the outcome of treatment, facilitating a more specific approach to therapy.

In this report, we have demonstrated a significant association between TGFBI expression and the success of chemotherapy. We evaluated 47 NSCLC clinical samples and we detected low levels of TGFBI in those patients who did not respond to chemotherapy. In contrast, in patients who did respond to chemotherapy, we found higher levels of this protein in their tissue samples, suggesting a possible role of this molecule as a surrogate marker for response to therapy.

TGFBI was described as a soluble TGFβ1-induced ECM binding protein; it mediates cell adhesion to extracellular proteins such as collagen, fibronectin and laminins through integrin binding [9]. Cell adhesion to ECM has been consistently reported as one of the mechanisms used by tumor cells to resist chemotherapy [28,29]. In this process, the ECM proteins play a critical role [30]. Here, in contrast to what was expected, we demonstrated that TGFBI silencing increased cell viability and over-expression had the opposite effect. Additionally, we observed an increase in chemo-sensitivity to a variety of chemotherapeutic agents when cells over-expressed TGFBI. This finding indicates that TGFBI promotes cell death through a mechanism that is independent of the drug used. Ahmed et al., suggested that TGFBI-mediated apoptosis was a direct effect of TGFBI on microtubule stabilization [22], and in our case we observed a direct caspase activation after TGFBI exposure regardless of using microtubule targeted therapy or other chemotherapeutic drugs. In spite of an apparent discrepancy, both mechanisms could be occurring at one time: full-length TGFBI could promote microtubule stabilization while its proteolytic fragments induce cell death.

We also demonstrated that TGFBI binds to NSCLC cells through the αvβ_3 _integrin. Considering the variety of integrin receptors that TGFBI protein can bind to, the specific identification of αvβ_3 _integrin as the key receptor highlights the pleiotropic nature of TGFBI-integrin signaling. In fact, TGFBI-mediated cellular response may vary widely depending on the integrin this protein binds to and the tissue location of this interaction. For example, TGFBI-αvβ_5 _integrin binding on vascular smooth muscle cells leads to increased adhesion and migration via Akt, ERK and FAK phosphorylation [31]; in other cells such as keratinocytes and corneal epithelial cells, TGFBI-mediated cell adhesion and migration is achieved through its binding with α_3_β_1 _integrin [32,33]. Interestingly, it has been described that TGFBI promoted anti-angiogenic responses in HUVEC cells through binding to the αvβ3 integrin [34], which is the same integrin that mediates TGFBI adhesion to ECM in response to acute hypoxia in lymphatic endothelial cells [35]. In our experiments, we observed that TGFBI binds to αvβ_3 _integrin on NSCLC cells and blocking this interaction abrogates the pro-apoptotic effect of TGFBI.

Recent published studies have demonstrated that, while ligand-bound αvβ_3 _integrin activates cell survival pathways and suppresses pro-apoptotic signals, non-ligated αvβ_3 _integrin or integrins bound to soluble substrates actively promote apoptosis [23]. In our case, TGFBI mediated the susceptibility of NSCLC cells to chemotherapy and this may be the result of direct TGFBI induction of cell death through the binding of its proteolytic fragments (< 3 kDa) to the αvβ_3 _integrin. A number of previous studies have reported that high concentrations of TGFBI promoted the apoptosis of CHO and H1299 cells [24,36] via RGD peptides, which are the products of TGFBI proteolysis, similar to what has been described for other ECM molecules [37]. This aspect may be of clinical relevance given that soluble ligands for αvβ_3 _integrin have been used in pre-clinical animal models of melanoma and ovarian cancer [38,39]. Additionally, TGFBI-derived peptides could be used in the near future as adjuvants in lung cancer therapy. Questions such as how TGFBI-derived peptides will affect non-tumor cells, which protease catalyzes TGFBI proteolysis and in what instances this proteolysis is activated all warrant future investigation.

In summary, here we provide evidence of the usefulness of TGFBI as a predictor of the outcome of chemotherapy in NSCLC and highlight the role of proteolytic TGFBI fragments in αvβ3 integrin-dependent NSCLC cell death.

## Conclusions

In the current report we have demonstrated an association between TGFBI expression and the outcome of chemotherapy in patients with NSCLC. This protein has been described to be down regulated in NSCLC cells but, currently, little is known about its relationship with chemotherapy outcomes. Despite the remarkable development of targeted therapies for advanced NSCLC, little improvement in overall survival has been achieved. Therefore, the identification of predictive or surrogate markers of the response to chemotherapy would permit selection of patients who are most likely to positively respond to treatment [40].

We demonstrated that TGFBI-derived proteolytic fragments induced cell death when this protein was present at high concentrations in NSCLC cells. TGFBI-mediated cell death depended on the binding of TGFBI proteolytic fragments to αvβ_3 _integrin and the induction of caspase 8 and caspase 3/7. This pro-apoptotic effect occurred independent of the chemotherapeutic drug used. Therefore, the current findings offer new perspectives in the use of TGFBI-derived peptides as co-adjuvants for the development of new therapeutics.

## Materials and methods

### NSCLC patients and response criteria

To analyze NSCLC susceptibility to chemotherapy, a series of retrospective samples from 47 stage IV NSCLC patients were obtained prior to chemotherapy at the Clínica Universidad de Navarra between 2000 and 2006. Eligible patients needed to be 18 or older at the time of treatment, with a histological confirmed diagnosis of stage IV NSCLC, treated in our institution with a platinum-taxane combination regimen in a first-line setting and followed at least until tumor response assessment. In addition, sufficient tumor sample from original biopsy was required in order to perform immunohistochemical analysis. The records of 103 stage-IV NSCLC consecutive patients treated in our institution were initially selected. After evaluation, 22 of the 103 patients were excluded because chemotherapy was not administered in a first-line setting. Other 19 patients were not eligible due to unavailability of sufficient tumor sample to perform immunohistochemical analysis. Finally, 15 patients initially treated in our institution were lost during follow-up before tumor response was assessed and thus were also excluded. Therefore the final number of samples analyzed was reduced to 47 cases.

Clinico-pathological features of these 47 patients are shown in Additional file 2. Histological diagnosis was performed according to the WHO classification [41]. REMARK criteria were followed throughout all the study [42]. Criteria for response were established as already published following RECIST criteria [43]. Complete response was defined as the disappearance of all target and non-target lesions, and the normalization of tumor marker level confirmed by repeat assessments that should be performed no less than 4 weeks after the criteria for response is first met. During this time no new lesions should appear. Partial response was defined as a greater than or equal to 30% decrease in the sum of the largest one-dimensional measurements, maintained for a minimum of 4 weeks. Stable disease was defined as change in the sum of the products or diameters, respectively, insufficient for partial response and progressive disease, maintained for a minimum of 4 weeks from baseline [44]. The study protocol was approved by the ethics committee.

### Immunohistochemistry of clinical biopsies from NSCLC patients

Formalin-fixed paraffin-embedded tissue sections were evaluated. Endogenous peroxidase activity was quenched and antigen retrieval was carried out by pressure cooking. Non-specific binding was blocked using 5% normal goat serum in Tris-buffered saline (TBS) for 30 min. Sections were incubated with anti-TGFBI antibody (1:25; Proteintech group, Chicago, USA) overnight at 4°C. Sections were then incubated with Envision polymer (Dako, Glostrup, Denmark) for 30 min at room temperature. Peroxidase activity was developed using diaminobenzidine and counterstained with hematoxylin before mounting in DPX mounting medium (BDH Chemical, Poole, UK). The specificity of the antibody was assessed by Western blot analysis of proteins from lung tumor sections (Additional file 1). Negative controls were carried out by omission of the primary antibody or incubation with an isotype control antibody.

### Immunostaining evaluation

Two independent, blinded observers (M.J.P and M.I.) evaluated the intensity and extensiveness of staining in all of the study samples. The evaluation of TGFBI expression was performed using the H-score system [45]. Briefly, the percent of positive cells (0-100%) was scored and the intensity of staining was assessed in comparison to an external positive control (1^+^, mild; 2^+^, moderate and 3^+^, intense labeling). A final H score (0-300) was established by multiplying the percentage of labeled cells and the intensity of staining. Disagreements were resolved by common re-evaluation.

### Cell culture

Human NSCLC cell lines derived from adenocarcinoma (NCI-A549) and large cell carcinoma (NCI-H1299) were purchased from American Type Culture Collection (ATCC, LGC-Promochem SL, Barcelona, Spain) and grown in RPMI 1640 medium (GIBCO, Barcelona, Spain) supplemented with 10% FBS and penicillin-streptomycin at 100 U/mL each. Cell cultures were incubated at 37°C in a humidified 5% CO_2 _incubator.

### TGFBI over-expression and silencing

To over-express TGFBI in H1299 cells (H1299 TGFBIve), 2 × 10^5 ^cells/well were transfected with 1 μg of the pCMV6-XL4 plasmid vector containing TGFBI cDNA (Origene, MD, USA) using 2 or 3 μl of FuGENE 6 Transfection Reagent (Roche, IN, USA) in 97 μl of Opti-MEM medium (Invitrogen, Barcelona, Spain) following manufacturer's instructions.

To silence TGFBI expression, 1 × 10^6 ^A549 cells/mL (A549-TGFBIsi) were transfected by electroporation with 10 μg of a commercially available shRNA targeting TGFBI cloned in a pRS plasmid vector (Origene, MD, USA). Cell transfections were performed in Opti-MEM medium (Invitrogen) using a Biorad Gene Pulsar I electroporator (Hercules CA, USA), with the capacitance set at 100 F and the voltage at 500 V. Transfection efficiency was confirmed by Western blot of NSCLC supernatants (Additional file 4).

### Determination of cell viability

For cell viability assays, 10^4 ^NSCLC cells were plated in 96-well plates (TPP, St. Louis MO, USA) in complete RPMI medium as described [46]. To measure the role of TGFBI expression in NSCLC sensitivity to chemotherapy, A549 TGFBIsi and H1299 TGFBIve cells were seeded in 96-well plates. When cells reached confluence, increasing amounts of the four different chemotherapeutic drugs were added to NSCLC cells in complete cell culture media for 48 h and cell viability was assayed. The IC_50 _for etoposide in non-transfected cells was determined experimentally and used to measure caspase 3/7 activity and PARP cleavage (50 μM for H1299 cells and 5 μM for A549 cells).

To determine the influence of rh-TGFBI protein on cell viability, soluble recombinant human TGFBI (rh-TGFBI) purchased from R&D, was added to the cells in increasing concentrations (0.1, 1, 5, 10 and 20 μg/mL) and cell viability was assayed after 24 h.

### Cell adhesion assay

To determine which integrin is involved in NSCLC cell adhesion to TGFBI, 1 × 10^6 ^cells/mL were labeled with 10 μM calcein-AM (Fluka, Steinheim, Germany) in adhesion-medium (RPMI 0.5% BSA, 20 mM HEPES). Cells in suspension were incubated with gentle rocking for an hour in the presence 1 μg/mL of each monoclonal anti-integrin blocking antibody. Afterwards, 10^4 ^cells were seeded onto 1 μg/mL rh-TGFBI coated wells, and cell adhesion was assayed as described [46]. Six replicates were plated for each condition. The following blocking monoclonal antibodies were used: anti-α_1 _integrin (MAB1973Z, Chemicon International, CA, USA), anti-β_3 _integrin (MAB2023Z, Chemicon International), anti-α_4 _integrin (340976, Becton Dickinson, NJ, USA), anti-integrins αv, β_1_, β_2_, α_5_β_1_, αvβ_3_, αvβ_5 _(Integrin Classics Kit, ECM435, Chemicon Interational) and a nonspecific IgG (MAB004, R&D Systems).

### PARP cleavage detection by Western blot analysis

To determine the effect of TGFBI expression on PARP cleavage in the NSCLC cells in response to chemotherapy, 10^6 ^NSCLC cells were cultured in RPMI complete medium on 10 cm^2 ^plates and allowed to adhere for 24 h. After this time, etoposide was added at the IC_50 _(50 μM for H1299 cells and 5 μM for A549 cells) for 48 h. Then, protein extracts were collected and transferred to nitrocellulose membranes as described [46]. The membranes were blocked by incubating them overnight in a solution of 5% non-fat dry milk in PBS-Tween. Primary PARP antibody (Cell Signaling, MA, USA) incubation was performed for 1 h at room temperature (1:1000 dilution). Membranes were washed three times with PBS-Tween 0.2% and incubated with an HRP-conjugated anti-rabbit IgG (sc-2030, Santa Cruz Biotechnology, CA, USA) in 5% non-fat dry milk in PBS-Tween. The signal was developed using the Lumi-light^PLUS ^Western Blotting Kit (Roche Molecular Biochemicals, Mannheim, Germany).

### Integrin αvβ_3 _flow cytometry

For determination of integrin αvβ3 expression on the surface of NSCLC cells, 1 × 10^5 ^cells A549 and H1299 cells were cultured to subconfluency in 6 well plates containing 1 mL of complete cell growth media. Afterwards, the cells were harvested and resuspended in 100 μl of staining buffer (5% FBS, EDTA 2 mM in PBS) and incubated for 10 min at room temperature with a concentration of 1 μg/ml of the, anti-integrin αvβ3 (Integrin Classics Kit, ECM435, Chemicon Interational) monoclonal antibody or a non-specific IgG (MAB004, R&D Systems). Afterwards, the cells were washed with staining buffer and subsequently incubated with 1:500 green-fluorescent Alexa Fluor 488 anti-mouse-secondary antibodies (Molecular Probes) at room temperature for 10 min. Cells were washed twice with staining buffer and analyzed on a FACScan flow cytometer using Cellquest software (BD-PharMingen).

### Caspase 3/7 and caspase 8 determination

To determine caspase 3/7 activity, 10^4 ^cells were cultured in RPMI complete medium on 96-well plates coated with increasing amounts of rh-TGFBI (rhTGFBI) and allowed to adhere for 24 h. After this time caspase 3/7 activity was measured. In some instances, the cells were allowed to adhere to non-coated wells for 8 h and then incubated with increasing amounts of rh-TGFBI for 24 h. To measure caspase 3/7 activity after etoposide treatment, NSCLC cells were incubated with this drug at the IC_50 _(50 μM for H1299 cells and 5 μM for A549 cells) for additional 24 h after being treated with or seeded onto rh-TGFBI, and caspase activity was measured afterwards.

For αvβ3 integrin blocking experiments, 10^4 ^cells were cultured in RPMI complete medium on 96-well plates and always allowed to adhere to non-coated surfaces for 8 h. After this time, the cells were pre-incubated for 1 h with 1 μg/mL of the monoclonal anti-αvβ3 integrin antibody (Chemicon, IL, USA). Then, 20 μg/mL of rh-TGFBI was added to the cells without removing the anti-integrin monoclonal antibody. When indicated, 24 h latter, etoposide was added at its IC_50 _for additional 24 h. Caspase 3/7 activity was estimated using the Caspase-Glo 3/7 Assay (Promega, Madrid, Spain) and quantified on a Polar Star Galaxy plate reader (BMG, Offenburg, Germany), using 530 emission filters. Three replicates were assayed for each condition.

To analyze the effects of RGD peptides on the apoptosis of NSCLC cells, we collected supernatants from A549 and H1299 cultures after cells were transiently transfected with the TGFBI expression vector. The amount of TGFBI protein in the cell supernatants was estimated by densitometric analysis of Western-blot bands using as reference, the intensity of 10 ng of recombinant protein loaded in the first lane of each gel. NSCLC cell supernatants were collected 24 h later and filtered through a 3 kDa exclusion column (Centricon, MA, USA). Both the concentrate (TGFBI < 3 kDa) and the filtrate (TGFBI > 3 kDa) were added to different A549 and H1299 cultures at a final concentration of 1× for 4, 8, 12 or 24 h. At each time-point, caspase 3/7 and caspase 8 activity were measured using the Caspase-Glo Assay (Promega) as above.

### Statistical analysis

Non-parametrical Kruskal-Wallis and pairwise Mann-Whitney U tests were used to compare TGFBI expression in NSCLC samples according to response to treatment. To asses overall survival, Kaplan-Meier curves were calculated and comparison was performed by log-rank test. The median (H-score 150) was chosen as a cut-off point. Student *t*-test statistical analysis was performed to compare different treatments in the vitro experiments. All the analyses were performed using SPSS 15.0 (Chicago, IL). A p value less than 0.05 was considered statistically significant.

Figure legends for additional figures and additional Material and Methods are provided as additional files 8 and 9 respectively.

## Competing interests

The authors declare that they have no competing interests.

## Authors' contributions

MI and AR performed all the cell culture, cloning, transfection, viability, adhesion and caspase assays, data managing and writing of the manuscript. RP and MJP, contributed in the establishment of a tumor repository bank. MI and MJP performed all the histological staining and quantifications. JA, and MI performed the statistical studies IG-B, MP-S and MDL performed all the clinical contributions to this paper: patient diagnosis, anatomo-pathological tissue preparation, evaluation and diagnosis of the biopsies, patient treatment and follow up, and preparation of all the clinico-pathological data. ES contributed in all the experiments needed to response to reviewers: shRNA experiments, peptide treatments and flow cytometry analyses.

All the authors read and approved the final manuscript.

## Supplementary Material

Additional file 1 **additional figure 1**. TGFBI expression in samples derived from NSCLC patients. Click here for file

Additional file 2 **additional table 1**. Clinico-pathological features of the NSCLC patients analyzed. Click here for file

Additional file 3 **additional figure 2**. Kaplan-Meier analysis of TGFBI expression and overall survival in NSCLC patients. Click here for file

Additional file 4 **additional figure 3**. Quantification of basal TGFBI expression and that obtained after over-expressing or silencing TGFBI gene in NSCLC cells. Click here for file

Additional file 5 **additional figure 4**. Cell viability of NSCLC cells transfected with TGFBI sh-RNA or TGFBI-expression vectors. Click here for file

Additional file 6 **additional figure 5**. A TGFBI derived RGD peptide induced cell death while TGFBI mutant RGD peptide did not. Click here for file

Additional file 7 **additional figure 6**. Integrin β3 silencing in H1299 cells abrogates their response to TGFBI <3 KDa supernatants. Click here for file

Additional file 8 **Additional figure legends**. figure legends for additional figures 1-6. Click here for file

Additional file 9 **Additional Material and Methods**. detailed description of the methods used to generate additional figures. Click here for file

## Aknowledgements

We are grateful to Patricia Martín and Saray Garasa for technical assistance.

This work was funded through the UTE Project CIMA. MI is recipient of a fellowship from the Department of Education from the Government of Navarra (Spain) and JA is supported by a fellowship from the Instituto de Salud Carlos III, Ministry of Health (Spain).
