# Supplementary material for: TGFBI expression is associated with a better response to chemotherapy in NSCLC
Source: Mol Cancer. 2010 May 28;9:130. doi: 10.1186/1476-4598-9-130 (PMC2900244; doi:10.1186/1476-4598-9-130)
Supplement: Additional file 1 — additional figure 1. TGFBI expression in samples derived from NSCLC patients. [file 1476-4598-9-130-S1.PPT]

## Slide 1
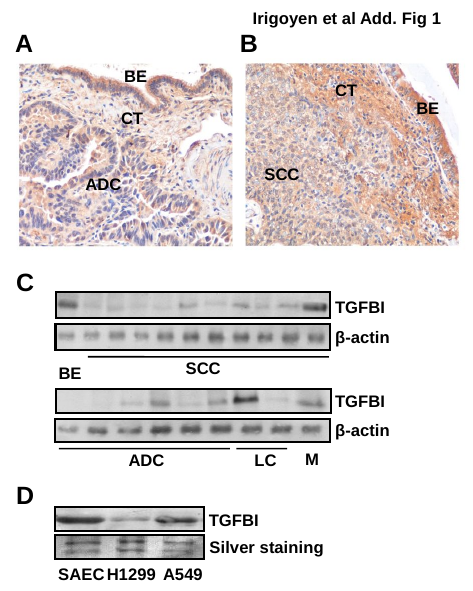

Irigoyen et al Add. Fig 1
A
B
BE
CT
BE
CT
SCC
ADC
C
TGFBI
β-actin
SCC
BE
TGFBI
β-actin
M
 LC
ADC
D
TGFBI
Silver staining
SAEC
H1299
A549
