# Supplementary material for: TGFBI expression is associated with a better response to chemotherapy in NSCLC
Source: Mol Cancer. 2010 May 28;9:130. doi: 10.1186/1476-4598-9-130 (PMC2900244; doi:10.1186/1476-4598-9-130)
Supplement: Additional file 2 — additional table 1. Clinico-pathological features of the NSCLC patients analyzed. [file 1476-4598-9-130-S2.DOC]

**Additional table 1: Clinico-pathological features of NSCLC patients**

| **Gender** | **Age** | **Tumor** | **Stage at** | **Procedence** | **Response to** | **TGFBI** | **Chemotherapy** |
| --- | --- | --- | --- | --- | --- | --- | --- |
|  |  | **histology** | **Chemotherapy** | **of tumor** | **treatment** |  | **Regimen** |
|  |  |  | **Initiation** | **biopsy** |  |  |  |
| Male | 56 | SCC | IV | Lung | Complete Response | 110 | PCG |
| Male | 57 | SCC | IV | Lung | Progression | 170 | PCb |
| Male | 66 | SCC | IV | Lung | Partial Response | 260 | PC |
| Male | 60 | SCC | IV | Bone | Partial Response | 180 | PCG |
| Female | 49 | ADC | IV | Pleura | Partial Response | 200 | PCG |
| Male | 63 | SCC | IV | Bronchus | Stabilization | 170 | PCG |
| Male | 50 | ADC | IV | Lymph-Node | Stabilization | 120 | PCG |
| Male | 57 | SCC | IV | Bronchus | Progression | 90 | PCG |
| Male | 69 | ADC | IV | Bone | Stabilization | 135 | PCG |
| Male | 60 | ADC | IV | Lymph-Node | Progression | 90 | PCG |
| Male | 67 | ADC | IV | Lymph-Node | Stabilization | 150 | PCG |
| Male | 68 | ADC | IV | Bronchus | Stabilization | 70 | PCG |
| Male | 67 | ADC | IV | Soft Tissue | Partial Response | 200 | PCG |
| Female | 63 | ADC | IV | Soft Tissue | Stabilization | 165 | PCG |
| Female | 48 | ADC | IV | Bronchus | Progression | 120 | PCG |
| Male | 54 | LCC | IV | Lung | Partial Response | 175 | PCG |
| Female | 45 | ADC | IV | Brain | Partial Response | 150 | PCG |
| Male | 62 | ADC | IV | Bronchus | Progression | 110 | PCG |
| Male | 50 | ADC | IV | Lung | Partial Response | 115 | PCG |
| Male | 57 | ALCC | IV | Lung | Partial Response | 160 | PCG |
| Male | 75 | Mixed | IV | Bone | Partial Response | 140 | PCG |
| Male | 56 | ADC | IV | Brain | Stabilization | 85 | PCG |
| Male | 57 | ADC | IV | Lung | Stabilization | 180 | PCG |
| Female | 45 | ADC | IV | Bronchus | Partial Response | 180 | PCG |
| Male | 62 | ADC | IV | Bronchus | Partial Response | 20 | PCG |
| Female | 59 | SCC | IV | Bronchus | Partial Response | 270 | PCG |
| Male | 42 | ADC | IV | Bronchus | Stabilization | 10 | PCG |
| Male | 60 | ADC | IV | Bronchus | Progression | 135 | PCG |
| Male | 61 | ADC | IV | Lymph-Node | Stabilization | 90 | PCG |
| Female | 77 | ADC | IV | Lung | Partial Response | 180 | PCG |
| Male | 52 | SCC | IV | Bronchus | Progression | 180 | PCG |
| Male | 62 | ADC | IV | Bronchus | Partial Response | 240 | PCG |
| Male | 74 | ADC | IV | Bronchus | Partial Response | 180 | PCG |
| Male | 54 | ADC | IV | Pleura | Progression | 70 | PCG |
| Female | 62 | ADC | IV | Bone | Stabilization | 70 | PCG |
| Male | 40 | Mixed | IV | Lung | Progression | 80 | PCG |
| Male | 50 | SCC | IV | Bronchus | Stabilization | 190 | PCG |
| Male | 62 | ADC | IV | Bronchus | Partial Response | 170 | PCG |
| Female | 56 | ADC | IV | Bronchus | Partial Response | 180 | PCG |
| Male | 54 | ADC | IV | Bronchus | Stabilization | 180 | PCG |
| Male | 50 | ADC | IV | Bronchus | Stabilization | 160 | PCG |
| Male | 60 | ADC | IV | Bronchus | Stabilization | 180 | PCG |
| Female | 55 | ADC | IV | Bronchus | Partial Response | 200 | PCG |
| Male | 53 | ADC | IV | Bronchus | Progression | 140 | PCb |
| Male | 55 | ADC | IV | Pleura | Progression | 50 | PCb |
| Male | 70 | SCC | IV | Bronchus | Partial Response | 110 | PCG |
| Male | 58 | ADC | IV | Bronchus | Partial Response | 160 | PC |

SCC, squamous cell carcinoma; ADC, adenocarcinoma; LCC, large cell carcinoma.

PCG, Paclitaxel-Cisplatin-Gemcitabine; PCb, Paclitaxel-Carboplatin; PC, Paclitaxel-Cisplatin.
