# Supplementary figures and images for: TGFBI expression is associated with a better response to chemotherapy in NSCLC
Source: Mol Cancer. 2010 May 28;9:130. doi: 10.1186/1476-4598-9-130 (PMC2900244; doi:10.1186/1476-4598-9-130)

## Slide 1
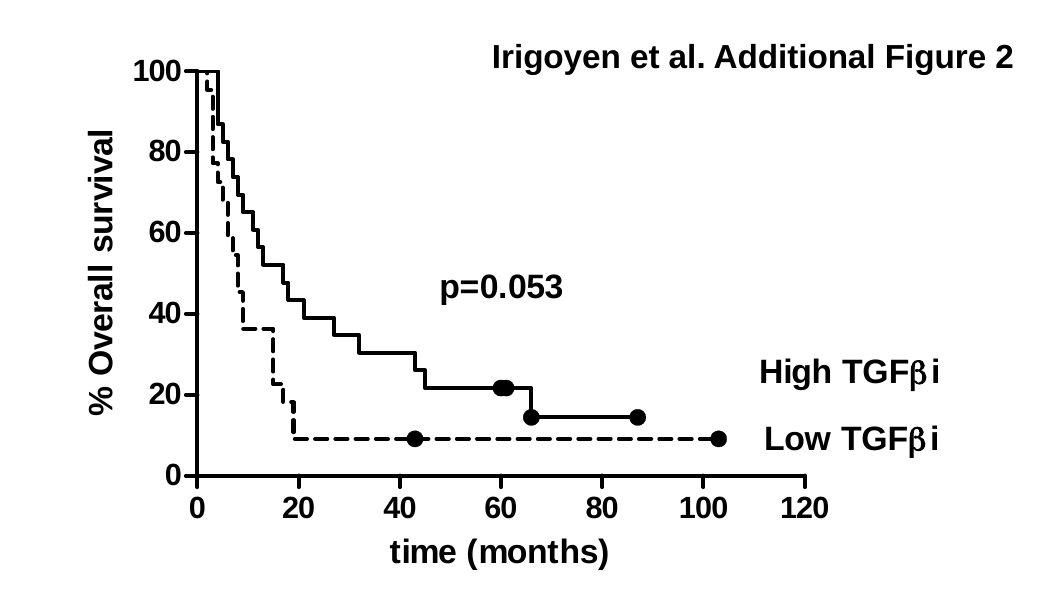

Irigoyen et al. Additional Figure 2

Supplement: Additional file 3 — additional figure 2. Kaplan-Meier analysis of TGFBI expression and overall survival in NSCLC patients. [file 1476-4598-9-130-S3.PPT]

## Slide 1
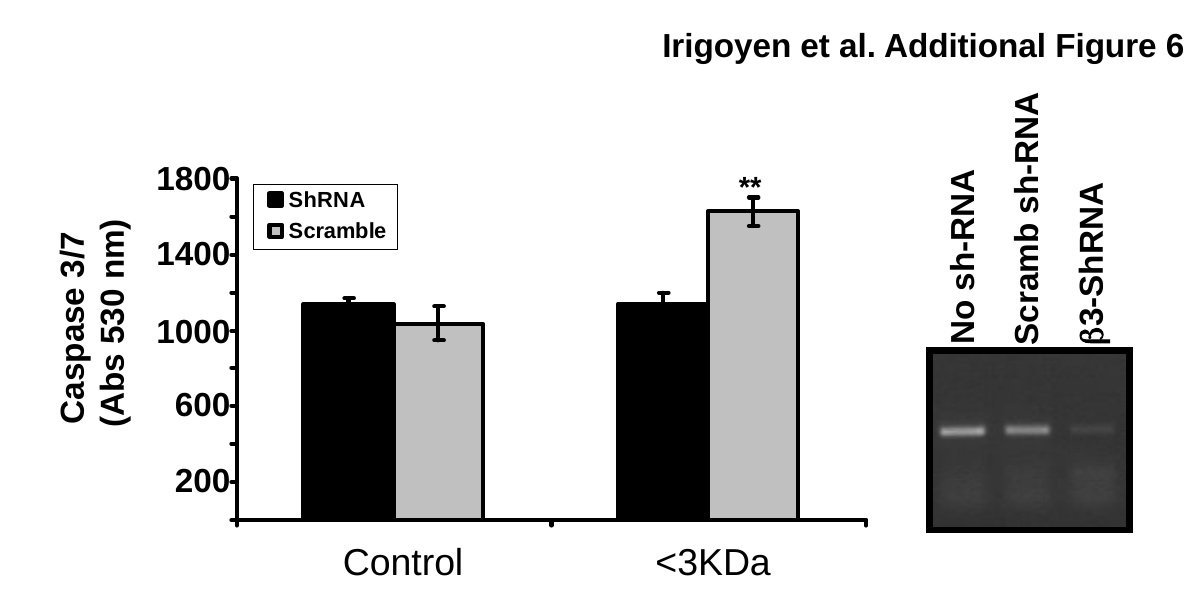

Irigoyen et al. Additional Figure 6
 Scramb sh-RNA
No sh-RNA
β3-ShRNA
1800
1400
Caspase 3/7
(Abs 530 nm)
1000
600
200
Control
<3KDa
**

Supplement: Additional file 7 — additional figure 6. Integrin β3 silencing in H1299 cells abrogates their response to TGFBI <3 KDa supernatants. [file 1476-4598-9-130-S7.PPT]
