# Supplementary material for: TGFBI expression is associated with a better response to chemotherapy in NSCLC
Source: Mol Cancer. 2010 May 28;9:130. doi: 10.1186/1476-4598-9-130 (PMC2900244; doi:10.1186/1476-4598-9-130)
Supplement: Additional file 4 — additional figure 3. Quantification of basal TGFBI expression and that obtained after over-expressing or silencing TGFBI gene in NSCLC cells. [file 1476-4598-9-130-S4.PPT]

## Slide 1
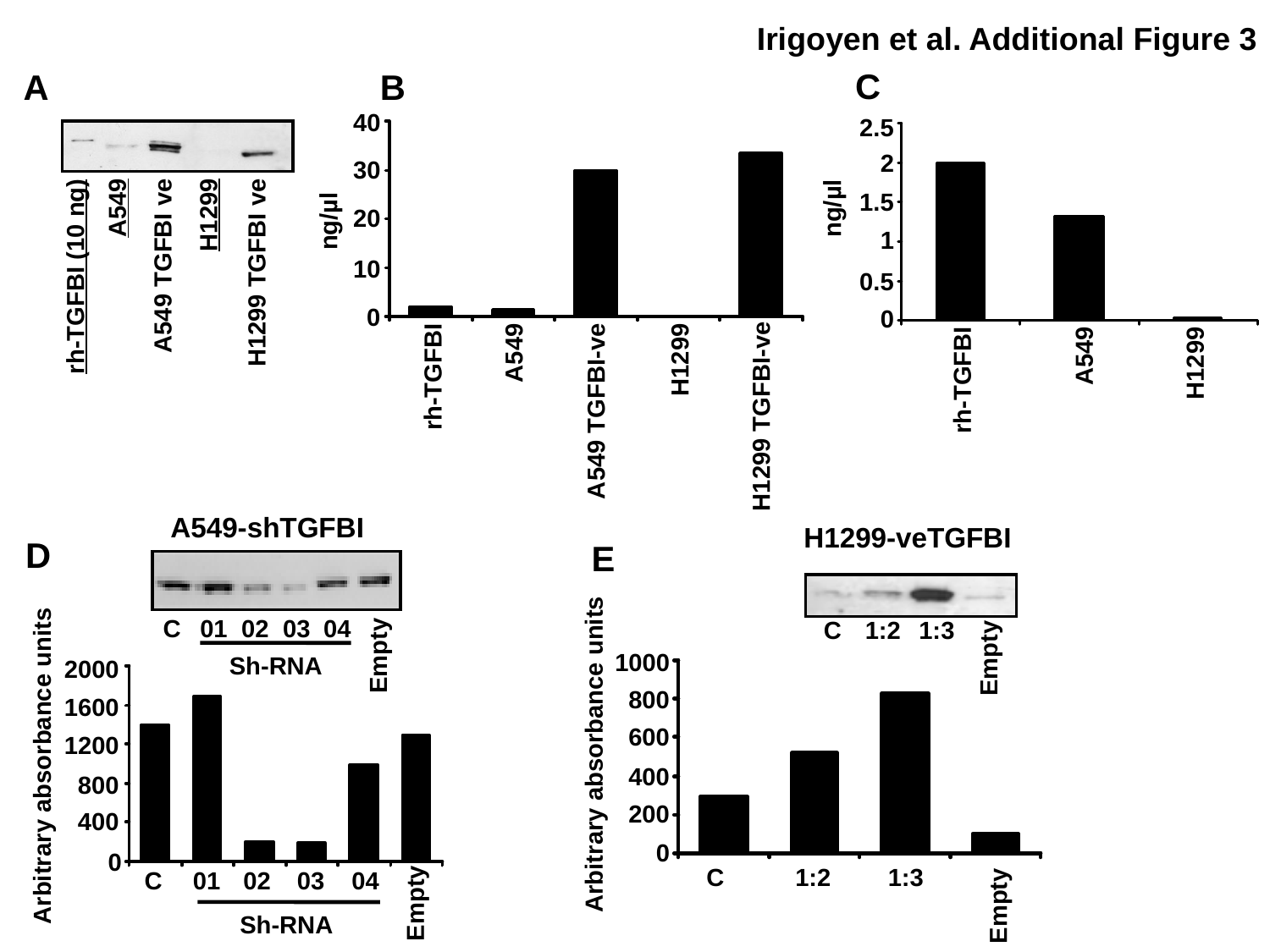

# Irigoyen et al. Additional Figure 3
C
A
B
40
30
20
ng/µl
10
0
A549
H1299
rh-TGFBI
A549 TGFBI-ve
H1299 TGFBI-ve
2.5
ng/µl
A549
H1299
rh-TGFBI
2
1.5
A549
H1299
1
A549 TGFBI ve
0.5
H1299 TGFBI ve
rh-TGFBI (10 ng)
0
A549-shTGFBI
H1299-veTGFBI
D
E
C
01
02
03
04
Empty
Sh-RNA
2000
1600
1200
800
400
0
C
01
02
03
04
Sh-RNA
Empty
C
1:2
1:3
Empty
1000
800
600
400
200
0
C
1:2
1:3
Empty
Arbitrary absorbance units
Arbitrary absorbance units
