# Supplementary material for: TGFBI expression is associated with a better response to chemotherapy in NSCLC
Source: Mol Cancer. 2010 May 28;9:130. doi: 10.1186/1476-4598-9-130 (PMC2900244; doi:10.1186/1476-4598-9-130)
Supplement: Additional file 5 — additional figure 4. Cell viability of NSCLC cells transfected with TGFBI sh-RNA or TGFBI-expression vectors. [file 1476-4598-9-130-S5.PPT]

## Slide 1
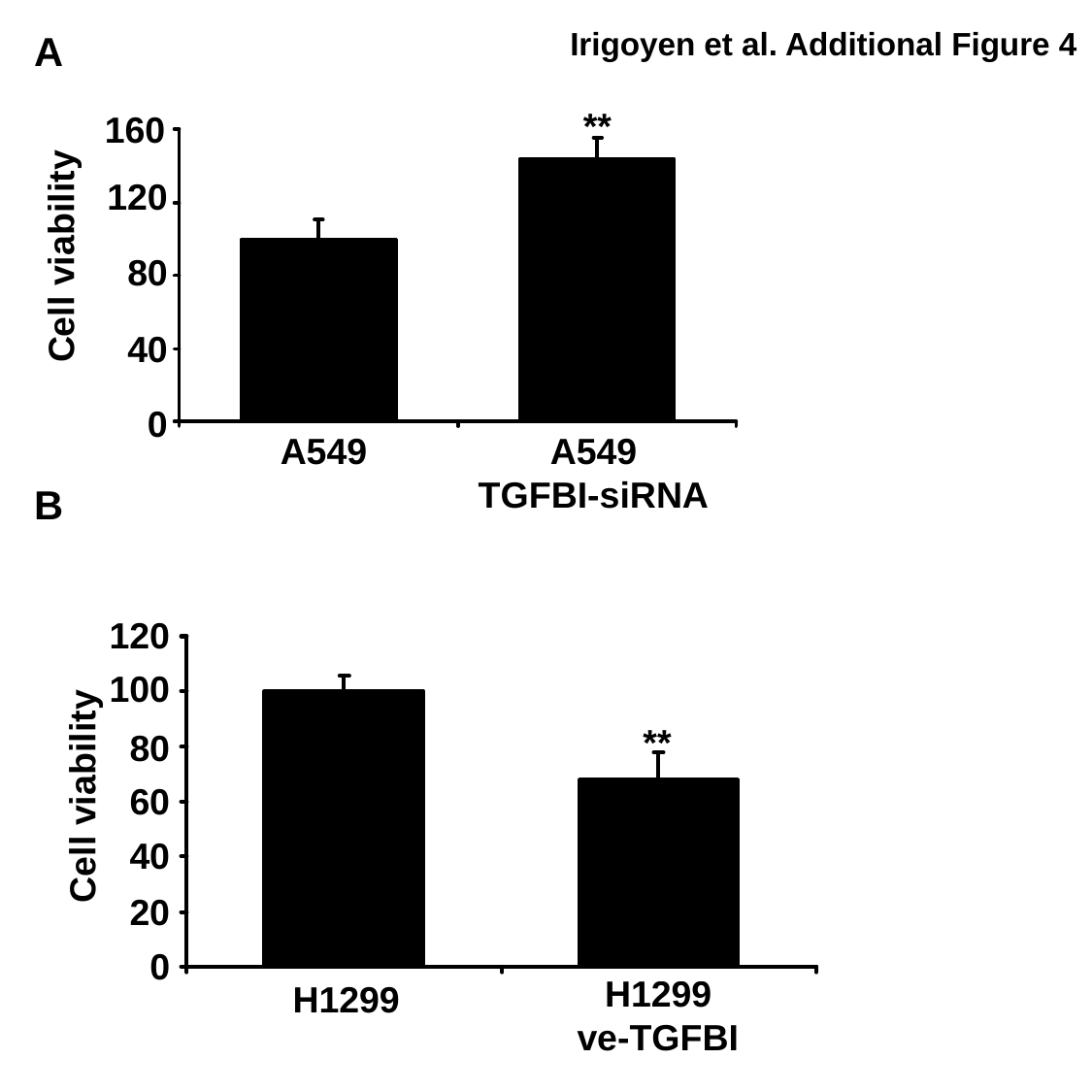

Irigoyen et al. Additional Figure 4
A
**
160
120
Cell viability
80
40
0
A549
A549
TGFBI-siRNA
B
120
100
**
80
Cell viability
60
40
20
0
H1299
ve-TGFBI
H1299
