# Supplementary material for: TGFBI expression is associated with a better response to chemotherapy in NSCLC
Source: Mol Cancer. 2010 May 28;9:130. doi: 10.1186/1476-4598-9-130 (PMC2900244; doi:10.1186/1476-4598-9-130)
Supplement: Additional file 6 — additional figure 5. A TGFBI derived RGD peptide induced cell death while TGFBI mutant RGD peptide did not. [file 1476-4598-9-130-S6.PPT]

## Slide 1
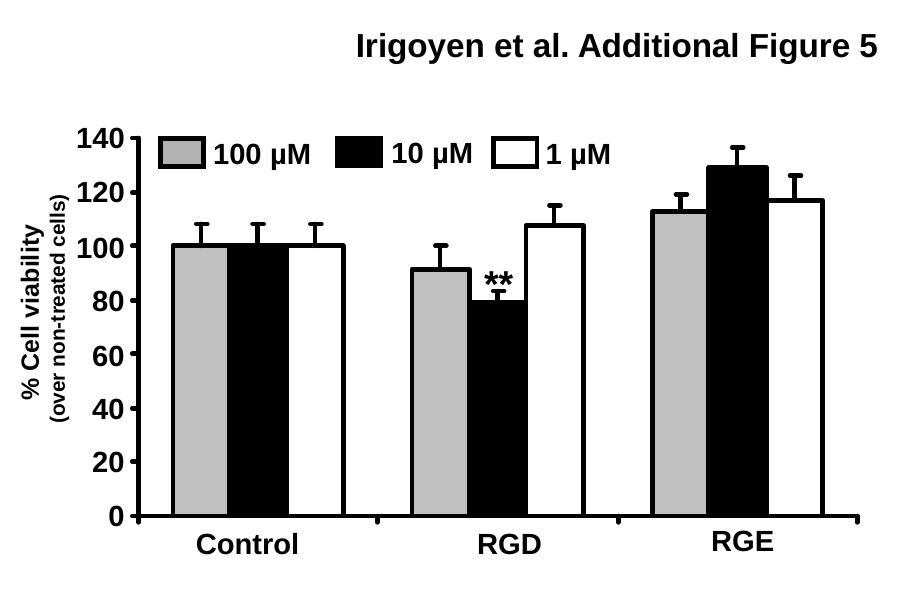

Irigoyen et al. Additional Figure 5
140
10 µM
100 µM
1 µM
120
100
% Cell viability
(over non-treated cells)
80
60
40
20
0
RGE
Control
RGD
**
