# Supplementary material for: TGFBI expression is associated with a better response to chemotherapy in NSCLC
Source: Mol Cancer. 2010 May 28;9:130. doi: 10.1186/1476-4598-9-130 (PMC2900244; doi:10.1186/1476-4598-9-130)
Supplement: Additional file 8 — Additional figure legends. figure legends for additional figures 1-6. [file 1476-4598-9-130-S8.DOC]

**Additional figure legends.**

**Additional figure 1. TGFBI expression in samples derived from NSCLC patients.** A representative example (from NSCLC patients) of TGFBI staining in adenocarcinoma (A) and squamous cell carcinoma (B) is shown. SCC, squamous cell carcinoma; ADC, adenocarcinoma, CT, connective tissue; BE, bronchial epithelium. C) Western blot analysis of TGFBI expression in protein extracts obtained from bronchial epithelium (BE), squamous cell carcinoma (SCC), adenocarcinoma (ADC), large cell carcinoma (LG) and mixed carcinoma (M). D) Western blot detection of TGFBI protein expression in the supernatant of A549 and H1299 NSCLC cells and in primary small airway epithelial cells (SAEC) purchased from Lonza.

**Additional figure 2. Kaplan-Meier analysis of TGFBI expression and overall survival in NSCLC patients.** Kaplan-Meier analysis of overall survival in a series of 47 patients of late stage NSCLC demonstrated a trend towards better survival in patients with high TGFBI expression (p=0.053).

**Additional figure 3. Quantification of basal TGFBI expression and that obtained after over-expressing or silencing TGFBI gene in NSCLC cells.** (A) Western-blot autoradiogram of basal expression of TGFBI and that obtained after transfection with the TGFBI expression plasmid TGFBI-pCMV6-XL4 (TGFBIve) in the A549 and H1299 NSCLC cells. 10 ng of rh TGFBI was loaded in the first lane of each gel. (B) Densitometry-based semi-quantification of TGFBI protein content in in the supernatants of non-transfected and TGFBIve transfected H1299 and A549 cells. (C) A detailed representation of TGFBI basal content in H1299 and A549 NSCLC cells as depicted in B. (D) TGFBI was silenced in the adenocarcinoma cell line A549 (A549-TGFBIsi) and overexpressed (E) in H1299 cells (H1299-TGFBIve) by transient transfection and validated by Western-blot analysis. Densitometry of the TGFBI specific bands were perfomed and depicted in the accompanying graphics. Two different DNA plasmid to FuGene ratios (1:2 and 1:3) were used to set up TGFBI overexpression, and four different sh-RNA plasmids against TGFBI were also tested. A ratio of 1:3 DNA/Fugene ratio and the shRNA 03 were selected for further studies.

**Additional figure 4. Cell viability of NSCLC cells transfected with TGFBI sh-RNA or TGFBI-expression vectors.** Viability of transfected NSCLC cells was determined 48 h after transfection with a TGFBI siRNA plasmid (A) or a TGFBI expression vector (B). Transfection with empty vector was used as a negative control for all the experiments. Three independent experiments were performed. Statistical comparisons of the differences between control and transfected cells were performed using Student *t*-test (***p*<0.01).

**Additional figure 5. TGFBI derived RGD peptides induced cell death while TGFBI mutant RGD peptides did not.** H1299 cells were exposed for 48 hours to different amounts of the TGFBI derived peptides: ERGDEL (RGD) and ERGEEL (RGE) in serum free media. Afterwards, cell viability was measured by the neutral red assay. Results from one representative experiment are shown as cell viability compared to that of untreated H1299 cells (100%). Statistical comparisons of the differences between control and treated cells were performed using Student *t*-test (***p*<0.01).

**Additional figure 6: Integrin β3 silencing in H1299 cells abrogates their response to TGFBI <3 KDa supernatants**. H1299 NSCLC cells were transiently transfected with anti-integrin β3-shRNA plasmids (shRNA) or a scrambled shRNA sequence. Integrin β3 silencing was determined by conventional PCR. Transfected cells were exposed for 48 h to TGFBI <3 KDa supernatants obtained from H1299 cells. Afterwards, caspase 3/7 activity was determined as stated in Materials and Methods. Statistical comparisons of the differences between control and transfected cells were performed using Student *t*-test (***p*<0.01).
