# Supplementary material for: TGFBI expression is associated with a better response to chemotherapy in NSCLC
Source: Mol Cancer. 2010 May 28;9:130. doi: 10.1186/1476-4598-9-130 (PMC2900244; doi:10.1186/1476-4598-9-130)
Supplement: Additional file 9 — Additional Material and Methods. detailed description of the methods used to generate additional figures. [file 1476-4598-9-130-S9.DOC]

**Additional Material and Methods:**

**TGFBI derived synthetic peptides induced cell death.**

The sequences of the TGFBI derived peptides were the same as published by Kim and cols. (See reference 26 of the manuscript). These peptides were synthesized and HPLC purified by JPT Peptide Technologies GmbH (Germany), dissolved in serum free media and added at the stated concentrations to 10^4^ NSCLC cells in serum free media. 48 hours latter cell viability was measured as determined in Material and Methods.

**Integrin β3 Isilencing sh-RNA experiments**

For Integrin β3 silencing, 10^6^ H1299 cells were transfected by electroporation with 10 µg of a commercially available shRNA targeting Integrin β3 cloned in a pRS plasmid vector (Origene, MD, USA). Cell transfections were performed in Opti-MEM medium (Invitrogen) using a Biorad Gene Pulsar I electroporator (Hercules CA, USA), with the capacitance set at 996 F and the voltage at 250 V. Cell transfection efficiency was tested by conventional RT-PCR (supplementary figure 6). 48 hours after transfection the cells were exposed to 1X concentrations of TGFBI <3kDa derived supernatants for additional 48 hours and caspase 3/7 activity was measured as stated above.
